# Secular Trends of Physical Fitness in Twenty-Five Birth Cohorts of Slovenian Children: A Population-Based Study

**DOI:** 10.3389/fpubh.2020.561273

**Published:** 2020-10-19

**Authors:** Žan Luca Potočnik, Gregor Jurak, Gregor Starc

**Affiliations:** Laboratory for Physical and Motor Development Diagnosis, Faculty of Sport, University of Ljubljana, Ljubljana, Slovenia

**Keywords:** physical fitness, children, secular trends, cohort, SLOfit

## Abstract

In Slovenia, the national SLOfit surveillance system of the somatic and motor development of children and youth has been enabling researchers to observe the developmental trends of the entire population of school-aged children since 1987. The national database currently incorporates over 7.2 million sets of measurements of eight fitness tests and three anthropometric measurements. Since 1991, as in the rest of the world, in Slovenia, there is a common perception that the physical fitness of contemporary children is in decline and below the level of the physical fitness of the previous generation's childhood fitness. Our paper examines the trends of physical fitness in 26 birth cohorts of 7–10-year-olds. The analysis shows that the secular trends of physical fitness in boys and especially in girls have been positive and that the level of physical fitness of recent birth cohorts exceeds the national average of physical fitness of the 1989–2019 period. At the same time, the analysis reveals that the distribution of physical fitness has been changing from almost normal in the cohorts born in the first half of the 1980s, toward positively skewed in the subsequent cohorts born before the year 2000, and bimodal distribution in the later cohorts, indicating growing inequality and polarization of the motor development of children.

## Introduction

There is a common perception that the physical fitness of children has declined over the last few generations due to growing sedentary behavior, lack of habitual physical activity, and the easy availability of energy-rich food. This perception, however, is based on a great deal of anecdotal and lay speculation since the evidence, deriving from longitudinal cohort studies of secular trends in physical fitness is scarce and the existing evidence inconclusive. The assessments of the secular trends of physical fitness of children have been distinctly understudied and have been mostly derived from temporal studies, relying mostly on cross-sectional designs, usually comparing only individual or joint age-groups of children at two or three time-points, predominantly comparing data on relatively small samples of one or several age-groups, and using a variety of different test batteries (1–14). Some studies of secular trends have been focusing only on the components of in aerobic fitness (15–17), while others focused only on the components muscular fitness (18–22).

We were unable to identify any study that used the cohort design of population data for the assessment of the secular trends in general physical fitness, which is why there is a lack of evidence on the actual secular trends of distribution of physical fitness comparing one generation of children to the other at multiple time points.

When studying secular trends of physical fitness, it is not sufficient to observe only the changes in the central tendency but also the changes in its distribution among the observed groups. We were, however, unable to identify any study of secular trends in physical fitness that addressed the problem of secular changes in its distribution; this is mainly due to a lack of national monitoring systems for the development of physical fitness that could regularly provide population data or at least nationally representative large datasets on the annual level.

In recent years, Hungary (23), Portugal (24), and Finland (25) have introduced national monitoring systems for the assessment of children's somatic development and functional capacity, but several years of measurements are required before they will be able to assess the secular trends of children's physical fitness in at least a few age groups of schoolchildren, since none of the systems covers the entire age-span of the school-going population. In Slovenia, however, the SLOfit national surveillance system for somatic and motor development of children and youth, implemented in 1982 (26), already enables the analyses of secular trends of childhood physical fitness of several generations on the population level.

The purpose of this study is not merely to analyze temporal trends bit to assess the actual secular trends of general physical fitness of Slovenian children (between ages 7 and 10) in 26 birth cohorts, born from 1983 until 2008, on the population data of the 1991–2019 period. In addition, the analysis attempts to assess whether the statistical distribution of physical fitness remained normal throughout generations with the entire population experiencing similar trends, or the distribution became skewed due to growing differences, reflected in the polarization of results at both extremes.

## Materials and Methods

### Participants

**Figure 1:**
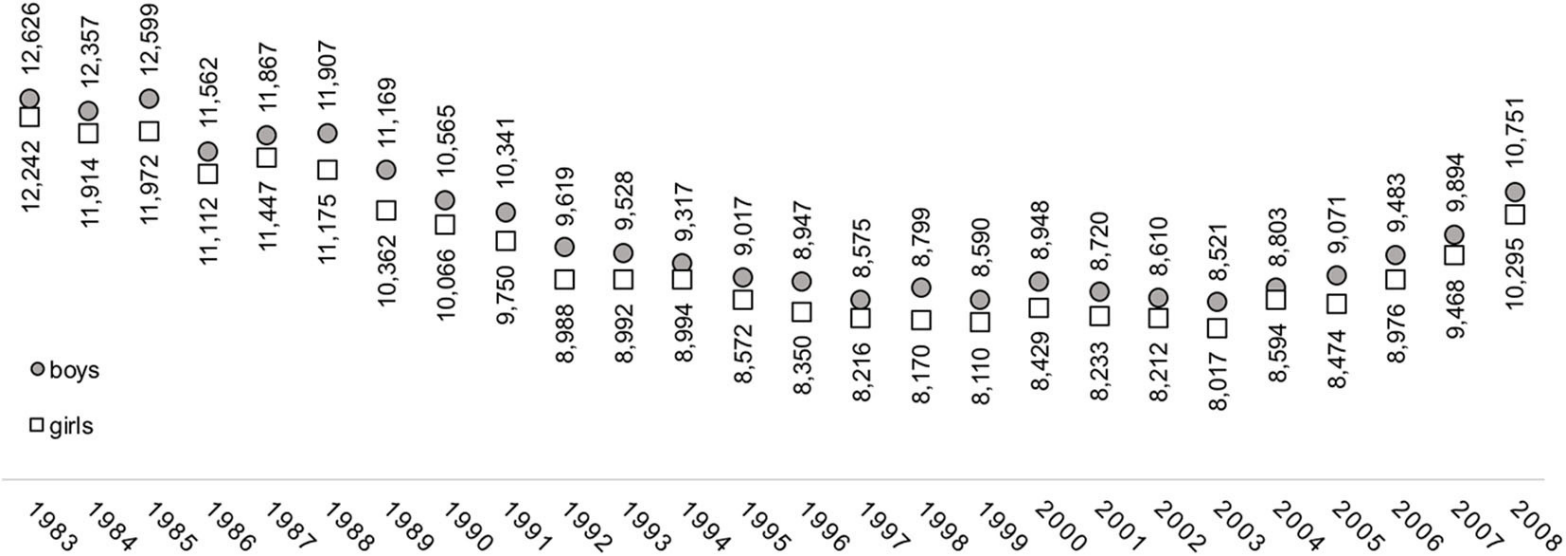
The number of boys and girls in the analyzed birth cohorts at age 10.

The analysis included 1,953,847 measurements of children (girls *n* = 953,001, boys *n* = 1,000,847) from 26 birth cohorts. Children from every birth cohort were measured annually at ages 7, 8, 9, and 10. The oldest cohort was born between January 1, and December 31 in 1983 and the youngest in 2008. In 2019, the youngest cohort was in primary school grade 5. The number of boys and girls in individual birth cohorts at age 10 are visible in Figure 1. After the breakup of Yugoslavia, Slovenia experienced a decrease in natality rates, which was reversed after 2003 and is also reflected in the number of children measured. The average response rate was around 94%, ranging from 88.7% in the 1986 birth cohort to 97.7% in the 2008 cohort. The difference in response rates between girls and boys was negligible and did not exceed 0.3% on the average.

### Instruments

#### Physical Fitness Assessment

The SLOfit test battery was used to assess the level of physical fitness of every child. This battery includes three anthropometric measurements (height, weight, and triceps skinfold thickness) and eight fitness tests (arm plate tapping, standing broad jump, backwards obstacle course, sit-ups in 60 s, stand and reach, bent-arm hang, 60-m dash, and 600-m run) (27).

Since each fitness test indicates a specific component of physical fitness, we used the Physical Fitness Index (PFI) based on the results of all fitness test as a single indicator of general physical fitness. A similar approach, utilizing a single indicator (Physical Fitness Indicator) based on the percentage of change of physical fitness, was utilized in two recent studies of physical fitness trends in Chinese populations (10, 13). In our case, PFI provides information about the percentile ranking of every child in the population of all Slovenian children in the 1989–2019 period (*N* > 7.2 million). To calculate the PFI, percentile scores of all eight fitness tests were calculated for each age-group, separate for boys and girls. Then, the mean percentile value of the eight fitness tests was calculated, each test contributing equally to PFI. The results of fitness tests were not adjusted to weight, height, triceps skinfold thickness, or BMI. The mean of eight fitness tests for every child was again ranked, and percentile score of PFI established on the entire population of the Slovenian children in the 1989–2019 period. In this way, the results of all individual tests for every child were ranked against the results of all children of same age and sex from 1989 onwards.

### Procedure

The SLOfit national surveillance system of somatic and motor development is a part of the Slovenian educational system. Its primary purpose is to provide teachers insight into the status of the somatic and motor development of their pupils to better plan and implement the PE lessons and other curricular and extracurricular sporting activities. All primary and secondary schools in Slovenia are required to organize and carry out annual assessments of the somatic and motor development for all children, with parental consent. The physical fitness tests are administered annually every April in the children's schools by physical education teachers according to the uniform national protocol and standardized equipment (27). All the schools in Slovenia send children's measurement results to the Laboratory for Physical and Motor Development Diagnosis at the Faculty of Sport, University of Ljubljana, where the data are cleaned, analyzed, and evaluated; feedback reports sent back to schools. The results of every child are rigorously checked using specially designed software, with all the ambiguities communicated back to the PE teachers and subsequently corrected. All PE teachers in Slovenia are educated at the Faculty of Sport and are thoroughly trained in the SLOfit measurements and data management, which are also regulated by the education legislation at the national level. In this way, the SLOfit system has been able to limit the individual influences of the observers on the results and sustain a high and homogeneous level of quality of data collection through the years.

The longitudinal cohort data with repeated measures were obtained from the SLOfit database to perform an exploratory analysis of secular trends of physical fitness. The selected age-span between 7 and 10 was used to minimize the effects of pubertal growth on physical fitness. Prior to the implementation of the 9-year primary school in the 1999/2000 school year, children in Slovenia had been entering primary school at age 7, but with the 9-year primary school, the admission age was changed to 6. The cohorts born before 1991, therefore, have data available only from age 7 onwards, which is why the data from age 6 was not included in the analysis.

### Statistical Analysis

The SPSS 26.0 for Mac statistical package was used to carry out the data analysis (Armonk, NY: IBM Corp.). The Kolmogorov–Smirnov test and Levene's test were used to determine the nature of the data. They provided scores below 0.05, whereby a decision was made to produce non-parametric statistics. The Generalized Linear Models (GLMs) method was used (28) to compare the secular trends of physical fitness in 26 birth cohorts. We used the AIC method of goodness of fit to construct the comparison model. GLMs with linear variable response distribution were selected, with model-based estimator of co-variance matrix. Wald Chi-square statistics with confidence interval type, and maximum likelihood estimation method was used to build the interaction model between the birth cohorts as a factor and PFI as dependent response variable to assess the secular trends in boys and girls. No tests for random effects were performed. Since PFI is a percentile value, calculated separately for every age and sex, we did not include the two variables in the model as covariates. Results were considered significant if *P* < 0.05. To assess the secular changes in the distribution of PFI in different birth cohorts, we produced histograms, and visually inspected the shape of distribution separately in boys and girls.

## Results

Tables 1, 2 show the statistics of the GLMs for boys and girls, respectively, which show significant differences in physical fitness between the youngest birth cohorts and the majority of older birth cohorts. They show that after the decline of physical fitness in the birth cohorts from the 1990s, the trends improved in the cohorts from the 2000s.

**Table 1:** GLMs statistics for boys.

**Birth cohort**	***B***	**SE**	**95% Wald CI**		**Hypothesis test**	
			**Lower**	**Upper**	**Wald Chi-Square**	**Sig**.
(Intercept)	51.057	0.1466	50.769	51.344	121,269.658	0.000
1983	−0.094	0.2046	−0.495	0.307	0.213	0.645
1984	−0.591	0.2044	−0.991	−0.190	8.352	0.004
1985	−0.925	0.2031	−1.323	−0.527	20.752	0.000
1986	−0.092	0.2063	−0.496	0.313	0.198	0.657
1987	−1.247	0.2048	−1.648	−0.845	37.064	0.000
1988	−0.850	0.2054	−1.253	−0.448	17.145	0.000
1989	−1.717	0.2086	−2.126	−1.308	67.755	0.000
1990	−1.830	0.2114	−2.244	−1.416	74.920	0.000
1991	−2.031	0.2123	−2.447	−1.615	91.479	0.000
1992	−1.391	0.2162	−1.814	−0.967	41.356	0.000
1993	−2.145	0.2169	−2.570	−1.720	97.795	0.000
1994	−2.454	0.2177	−2.881	−2.028	127.115	0.000
1995	−2.084	0.2183	−2.512	−1.657	91.178	0.000
1996	−2.387	0.2181	−2.814	−1.959	119.734	0.000
1997	−2.398	0.2194	−2.828	−1.968	119.427	0.000
1998	−2.893	0.2198	−3.324	−2.463	173.215	0.000
1999	−2.898	0.2205	−3.330	−2.466	172.747	0.000
2000	−3.095	0.2174	−3.522	−2.669	202.646	0.000
2001	−1.969	0.2192	−2.398	−1.539	80.695	0.000
2002	−2.368	0.2193	−2.798	−1.938	116.598	0.000
2003	−1.704	0.2200	−2.135	−1.272	59.936	0.000
2004	−0.747	0.2181	−1.175	−0.320	11.735	0.001
2005	−1.123	0.2166	−1.548	−0.699	26.900	0.000
2006	−0.256	0.2137	−0.675	0.163	1.432	0.231
2007	0.642	0.2113	0.228	1.056	9.226	0.002
2008	0^a^					

a *2008 is a reference cohort*.

**Table 2:** GLMs statistics for girls.

**Birth cohort**	**B**	**SE**	**95% Wald CI**		**Hypothesis test**	
			**Lower**	**Upper**	**Wald Chi–Square**	**Sig**.
(Intercept)	53.822	0.149	53.529	54.115	129,716.781	0.000
1983	−4.294	0.208	−4.701	−3.886	426.668	0.000
1984	−4.132	0.208	−4.540	−3.725	395.143	0.000
1985	−5.506	0.207	−5.913	−5.099	704.600	0.000
1986	−4.902	0.210	−5.314	−4.490	544.259	0.000
1987	−5.820	0.209	−6.229	−5.411	777.780	0.000
1988	−5.707	0.210	−6.119	−5.295	737.415	0.000
1989	−7.015	0.215	−7.436	−6.593	1,063.253	0.000
1990	−7.432	0.216	−7.856	−7.008	1,178.927	0.000
1991	−6.628	0.218	−7.055	−6.200	922.504	0.000
1992	−5.354	0.223	−5.790	−4.918	579.157	0.000
1993	−5.794	0.223	−6.230	−5.358	677.419	0.000
1994	−5.811	0.222	−6.246	−5.376	685.885	0.000
1995	−5.500	0.224	−5.939	−5.061	603.873	0.000
1996	−4.644	0.224	−5.083	−4.204	428.686	0.000
1997	−4.242	0.225	−4.683	−3.802	356.548	0.000
1998	−4.072	0.226	−4.515	−3.629	324.899	0.000
1999	−4.704	0.227	−5.149	−4.260	429.917	0.000
2000	−3.873	0.224	−4.312	−3.435	299.629	0.000
2001	−3.783	0.225	−4.224	−3.342	282.201	0.000
2002	−2.851	0.225	−3.291	−2.411	161.055	0.000
2003	−1.709	0.226	−2.151	−1.266	57.272	0.000
2004	−0.585	0.223	−1.021	−0.149	6.916	0.009
2005	−1.710	0.223	−2.147	−1.273	58.758	0.000
2006	−0.272	0.219	−0.702	0.158	1.534	0.215
2007	0.314	0.216	−0.110	0.738	2.109	0.146
2008	0^a^					

a *2008 is a reference cohort*.

**Figure 2:**
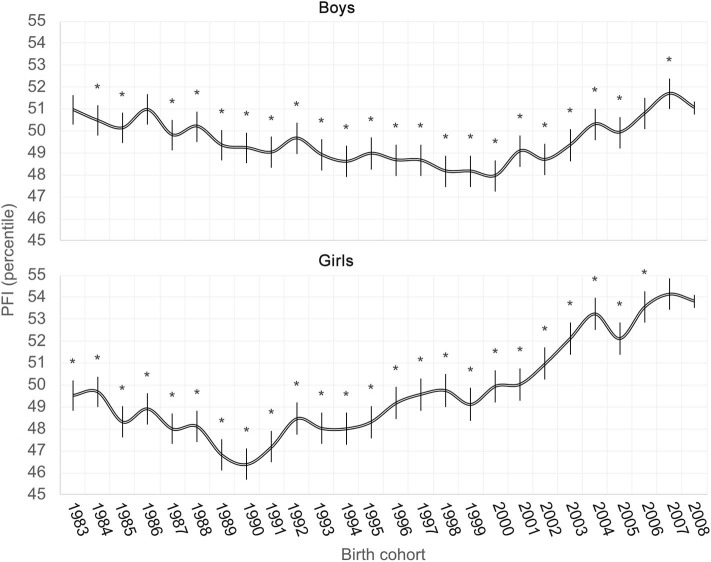
Secular trends of physical fitness in boys and girls (*significant difference from the reference birth cohort of 2008).

For boys, there was no significant difference in PFI between the 2008, 1983, 1986, and 2006 cohorts, while all other cohorts, except for the 2007 birth cohort, had significantly lower PFI than the 2008 reference cohort (*P* < 0.005) (Figure 2). The 2000 birth cohort in boys experienced the poorest development of physical fitness while the first cohort that again exceeded the average national PFI of the 1989–2019 period was the 2004 birth cohort.

For girls, the positive secular trend of PFI was much more pronounced and started improving in the 1991 birth cohort (Figure 2), but there were significant differences in PFI between the 2008 birth cohort and all other birth cohorts except for the cohort born in 2007 (*P* < 0.010). All the birth cohorts of girls until 2001 were below the average national PFI of the 1989–2019 period, but all younger birth cohorts achieved the level of PFI above the national average. The lowest level of PFI in girls was observed in the 1990 birth cohort.

**Figure 3:**
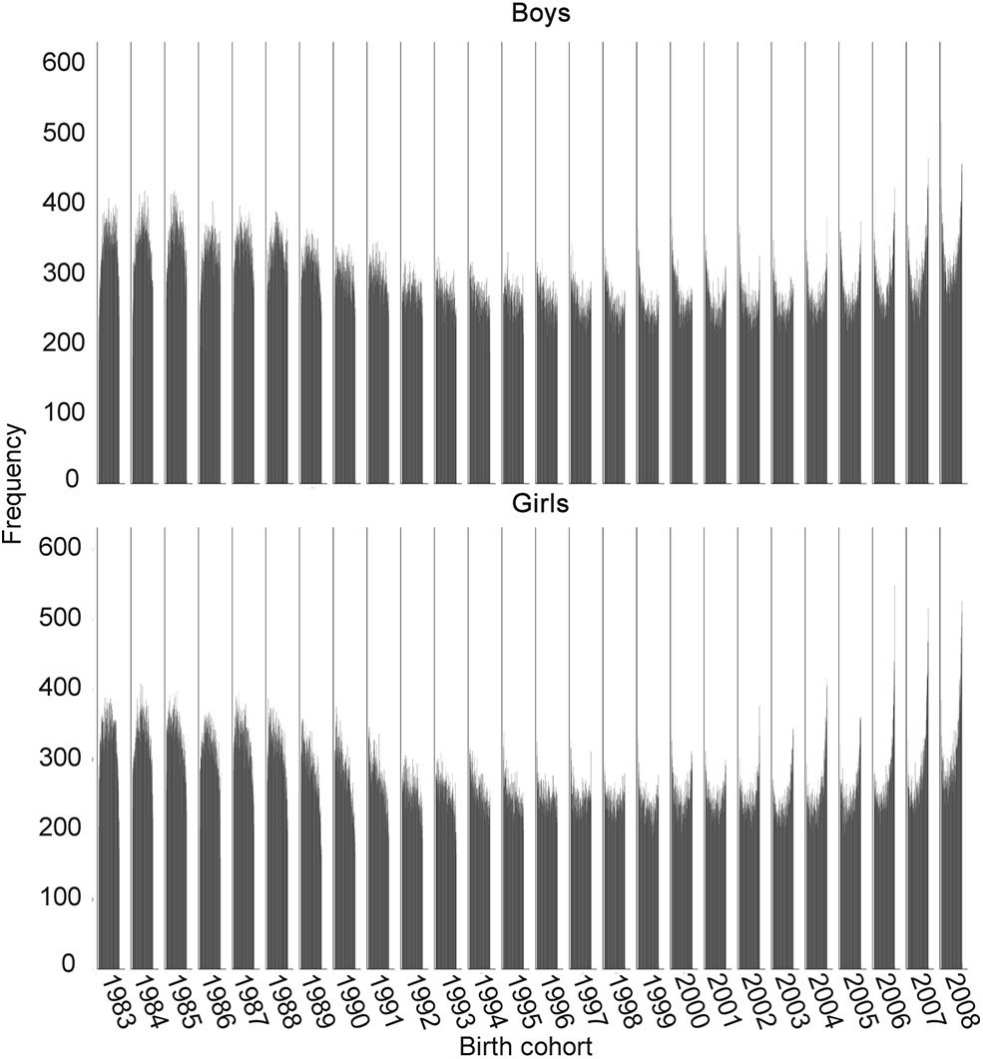
Distribution of PFI in boys and girls (mean percentile, CI, *significant difference from the reference birth cohort of 2008).

**Figure 4:**
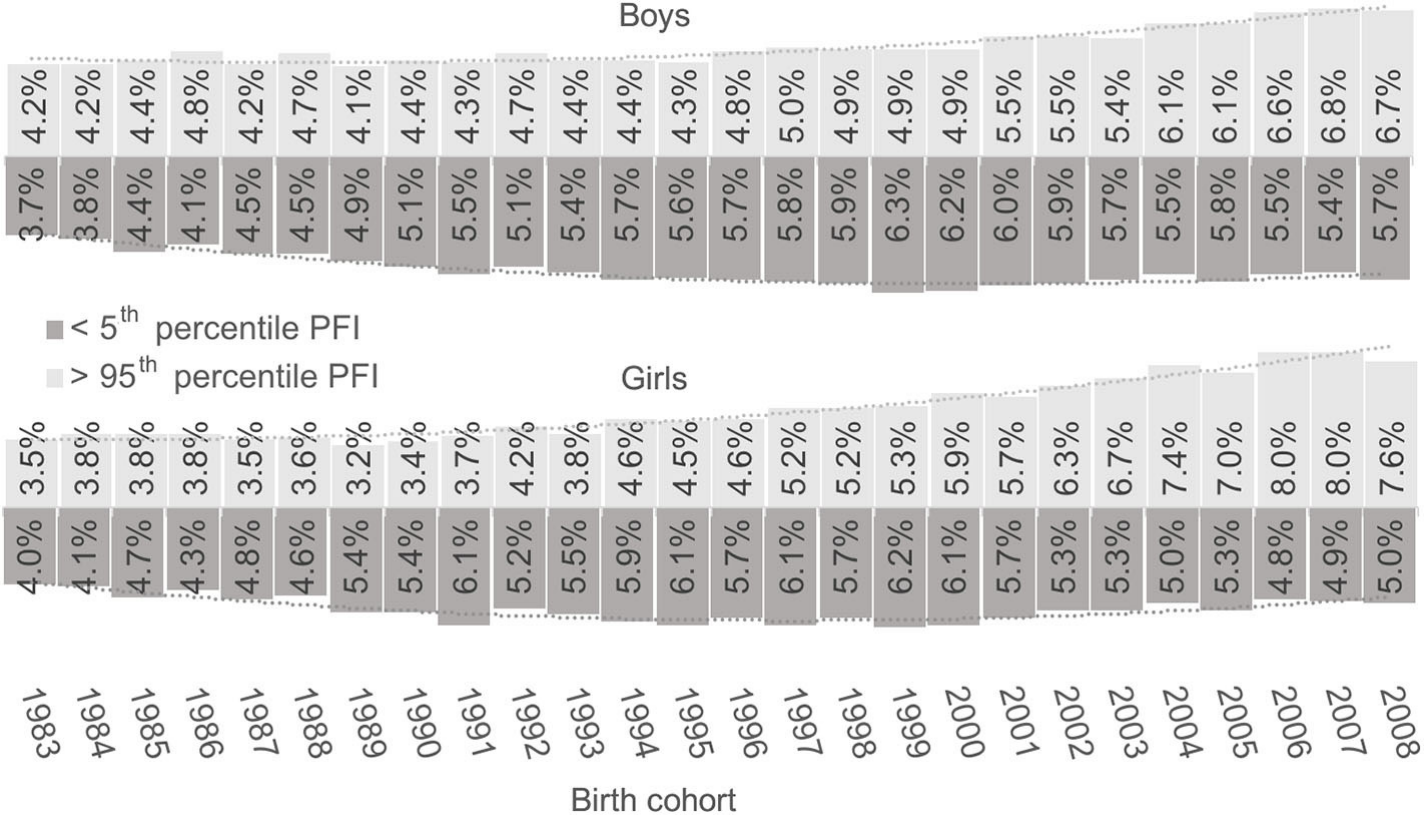
Share of children above 95th and below 5th percentile of PFI per birth cohort.

Although the analysis refutes the commonsensical speculations of declining general physical fitness and evidence about the positive secular trend, the changes in the distribution of PFI tell another interesting story (Figure 3). Namely, with every generation of boys and girls after 1983, the distribution of PFI deviated from the normal one toward the bimodal distribution with pronounced extremes, which evidence about a worrying trend of increasing inequality and polarization of physical fitness development in recent cohorts. More evidence of growing inequality is the growth of the share of children at both extremes of the distribution, specifically, those with poor physical fitness below the 5th percentile of the 1989–2019 period, and those with extraordinary physical fitness above 95th percentile (Figure 4). For boys and girls, the share of children with extremely low PFI was growing with every birth cohort until the year 2000 but has afterwards started to decline, although it is still higher than in the generations born in the 1980s. In contrast, the share of children with extremely high PFI has been growing with almost every generation, which has been especially expressed in girls. The share of girls with extremely high PFI in the most recent five generations has doubled in comparison with the 1983 generation, but overall, the overall share of boys and girls at both extremes rose by more than 70 per cent from 1983 to 2007 birth cohorts.

## Discussion

Our findings suggest that children in Slovenia have been experiencing positive trends of general physical fitness development, which was much more pronounced in girls than in boys. In this sense, our results do not reflect the negative global temporal trends of children's physical fitness (29, 30).

Furthermore, our findings suggest that the distribution of PFI in different birth cohorts changed from almost normal into bimodal due to growing differences in the development of children, evidencing that a part of the population did not experience the same favorable development.

The average level of general physical fitness in Slovenian children has been increasing with almost every generation of boys who were born since the year 2000, and especially with almost every generation of girls, born in the post-Yugoslav period from 1991 onwards, which is in concordance and probably related to the recent finding that the trends of overweight and obesity among Slovenian children and youth have been in decline from 2010 onwards (31).

Although we were unable to identify any other study that compared the secular trends of general physical fitness between different generations, the findings of our study show the opposite trends than the majority of recent studies of secular and temporal trends from other countries (4, 5, 7–11, 13, 18, 21). Nevertheless, we identified two recent studies that showed somewhat similar results to our analysis. A study among the Portuguese school children established the positive trend of certain components of physical fitness in the period between 1993 and 2013 (6), but the secular trends in our study are most similar to the trends among German children, who experienced a slight positive increase in numerous physical fitness components in the 2003–2017 period (14). The similarity of our results with the secular trends in Germany was also observed in the more expressed positive trend among girls, which was in opposition to the findings of the above-mentioned recent studies in which the trends of physical fitness were most often more negative among girls than boys.

Despite the established positive secular trends of physical fitness in Slovenia, the changes in the distribution of PFI suggest a growing inequality in the opportunities for physical activity and polarization of physical fitness in the population, which could be connected to socio-economic reasons as was already evidenced in the studies of Danish children (2, 15) in which children from different socio-economic conditions experienced different trends of development. The almost normal distribution of physical fitness in the Slovenian cohorts, born in the first half of the 1980s who spent their pre-school period of life without computers and other screen technology, was rapidly changing into a positively skewed distribution with every new generation, resulting in growing numbers of children with low levels of physical fitness and diminishing numbers of children with high levels of physical fitness. In the 2010/2011 school year, Slovenia launched a national intervention program, Healthy Lifestyle, which provided two additional lessons of physical education per week to more than 30,000 children and significantly improved the perception of physical activity as a prerequisite of healthy development in the general public and also in schools that were not involved in the program. In the last decade, a large number of schools in Slovenia have also implemented other forms of “super-standard PE,” resulting in increased time available for professionally guided physical activity within school settings. The Healthy Lifestyle program and other forms of additional PE were increasingly available to the cohorts of children born after the year 2000 and probably contributed to the improvement of physical fitness, but they did not add to the normalization of its distribution even if they have helped to improve the overall population median. In these cohorts, the positively skewed distribution of physical fitness became bimodal with a tendency toward negative skewness, evidencing the possibility to decrease the number of children with low physical fitness and increasing the number of children with excellent levels of physical fitness.

The methodology of our study differs substantially from the other existing studies of secular trends of physical fitness; it is also larger in scale and broader in scope than the previous studies, which make the direct comparison with other studies difficult but at the same time introduces a new perspective on the intra-generational change in physical fitness of children. By using a single indicator of physical fitness, incorporating most components of cardiorespiratory, muscular, and neuro-motor physical fitness, we attempted to surpass the limitations of looking only at a certain component of physical fitness. By looking at the physical fitness of individual birth cohorts throughout several years (7–10), we also attempted to avoid the limitations of looking only at a status of physical fitness of a cohort at only one time-point, which can be strongly influenced by individual differences in physical maturation. In comparison to the existing studies, our study does not focus only on the central tendency of the trend but also looks at the changes in the distribution of physical fitness, identifying a problem of growing inequality of physical fitness development.

The main strength of our study is its robustness due to the population data, its small probability bias due to high response rates, well-established test battery, highly qualified PE teachers performing the measurements, as well as its uniform measurement protocol and cohort design. However, it also has certain limitations. It uses one indicator of physical fitness as the aggregate of cardiorespiratory, muscular, and neuro-motor fitness, which can mask the negative trends in individual dimensions of physical fitness on behalf of positive trends in another dimension (for secular trends of individual fitness tests see Supplementary Figures 1–8). The analyzed data also do not enable the inclusion of various covariates that could reveal the causes of improved secular trends. The results are convincing and confirm that the physical fitness of children in Slovenia is improving, but they do not provide the answers about which environmental factors are the main cofounders and mediators of this improvement. Future research should, therefore, focus on studying the secular trends of physical fitness with different socio-economic indicators as covariates and should differentiate between children who were involved in interventions and sports training programs and those that were not. Although significant emphasis is placed on the assurance of the quality of data due to the high competence level of the PE teachers in measurement procedures, by standardized measurement conditions, and rigorous centralized data cleaning procedures, there are a large number of tests, a large number of schools, and a large number of children in a long period, which can compromise the quality of the data to some extent.

Lastly, the observed positive secular trends were studied only on the population of children between their ages 7 and 10 and do not provide information on the further development of their physical fitness in adolescence in adulthood.

## Conclusion

Our study attempted to assess the secular trends of physical fitness and changes in its distribution in 23 birth cohorts of Slovenian children. Our primary research questions attempted to determine whether the negative temporal trends, evidenced in the majority of other studies, have also been evident in the population of Slovenian children and whether the changes in the central tendency of physical fitness change were equally distributed throughout every cohort.

The PFI of Slovenian children, as an indicator of physical efficiency, encompassing cardiorespiratory, muscular, and neuro-motor fitness, shows that the secular trends of physical fitness of children in Slovenia are positive and that the recent generations of girls are experiencing strong emancipation through participation in regular physical activity and sport. Their level of physical fitness is superior to that of their mothers, born more than 20 years ago. The most recent cohorts of boys also managed to reach the physical fitness level of their fathers despite the unfavorable, sedentary environment in which they have been living. This positive trend is in contrast to observed temporal global trends (29, 30, 32) but simultaneously confirms that the contemporary generations of children in Slovenia are in fact among the most physically active children in the world (33) and have been also experiencing declining trends of overweight and obesity (31), which is directly reflected in the level of their physical fitness.

In contrast, the positive trend has not been experienced by the entire population of children, and with every generation, the share of children at the extremes of the distribution curve has been growing.

Our findings suggest that the high quality level of physical education teaching in Slovenia, supported by evidence data, exceptional school sports infrastructure, and additional hours of PE, provided by the Healthy Lifestyle intervention or at the initiative of schools, provided more favorable conditions for the development of physical fitness of children. However, the findings also suggest that the problem of growing inequality in physical fitness development should be considered and effective solutions found to provide better opportunities for development to the children at the negative end of the distributional curve.

## Data Availability Statement

The datasets presented in this study can be found in online repositories. The names of the repository/repositories and accession number(s) can be found below: http://www.slofit.org/Portals/0/Clanki/Datasets/Secular_trends_SLO_fitness_anon.zip?ver=2020-08-15-135026-590.

## Ethics Statement

The studies involving human participants were reviewed and approved by National Medical Ethics Committee of the Republic of Slovenia (ID 102/03/15). Written informed consent to participate in this study was provided by the participants' legal guardian/next of kin.

## Author Contributions

GS and ŽP conceptualized and designed the study, performed the statistical analysis, and wrote the first draft of the manuscript. ŽP organized the database. GJ wrote sections of the manuscript. All authors contributed to manuscript revision, read, and approved the submitted version.

## Conflict of Interest

The authors declare that the research was conducted in the absence of any commercial or financial relationships that could be construed as a potential conflict of interest.
