# Supplementary material for: Secular Trends of Physical Fitness in Twenty-Five Birth Cohorts of Slovenian Children: A Population-Based Study
Source: Front Public Health. 2020 Oct 19;8:561273. doi: 10.3389/fpubh.2020.561273 (PMC7604349; doi:10.3389/fpubh.2020.561273)
Supplement: Supplementary file 1 [file Data_Sheet_1.ZIP › Supplementary_Material.docx]

Supplementary Material


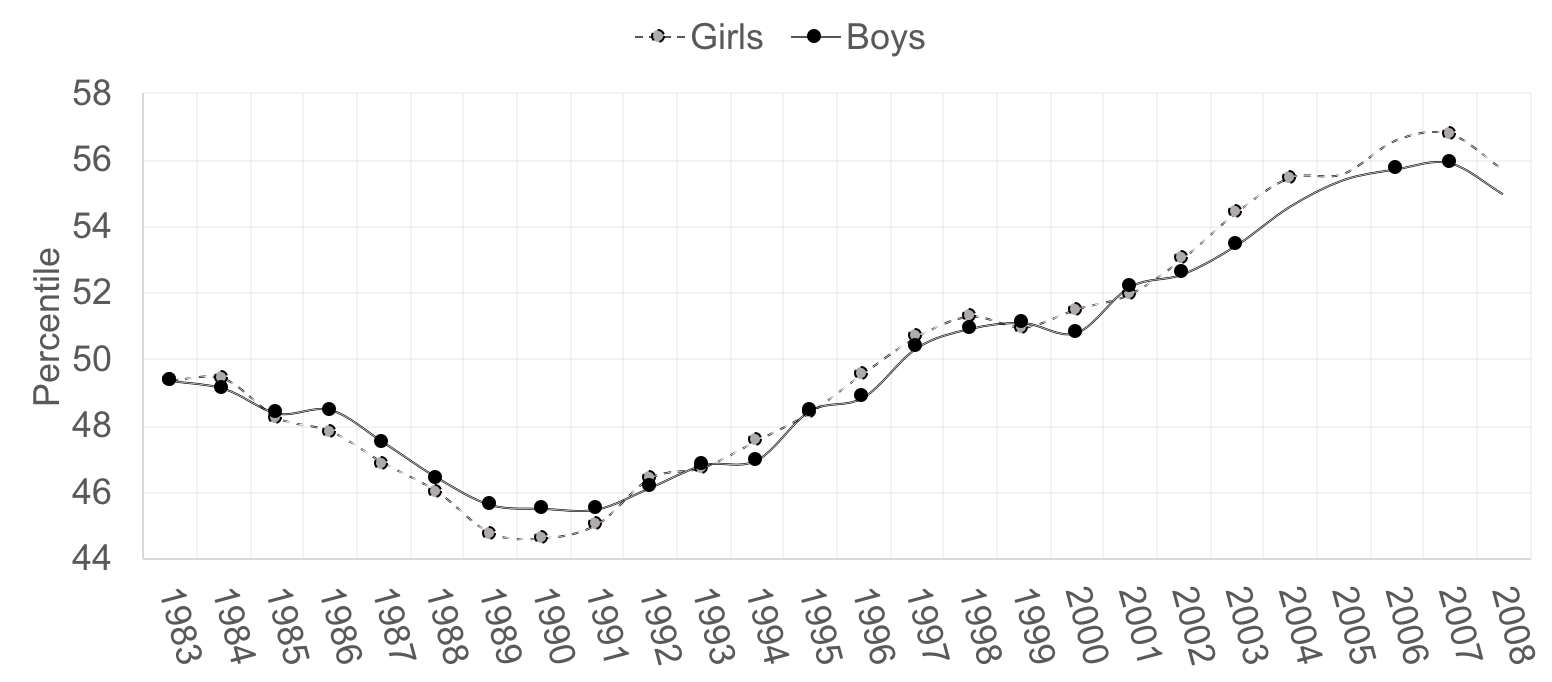


**Supplementary Figure 1.** Secular trends of the results of the arm plate tapping test by birth cohorts (2008 is the reference cohort and dots represent statistically significant differences)


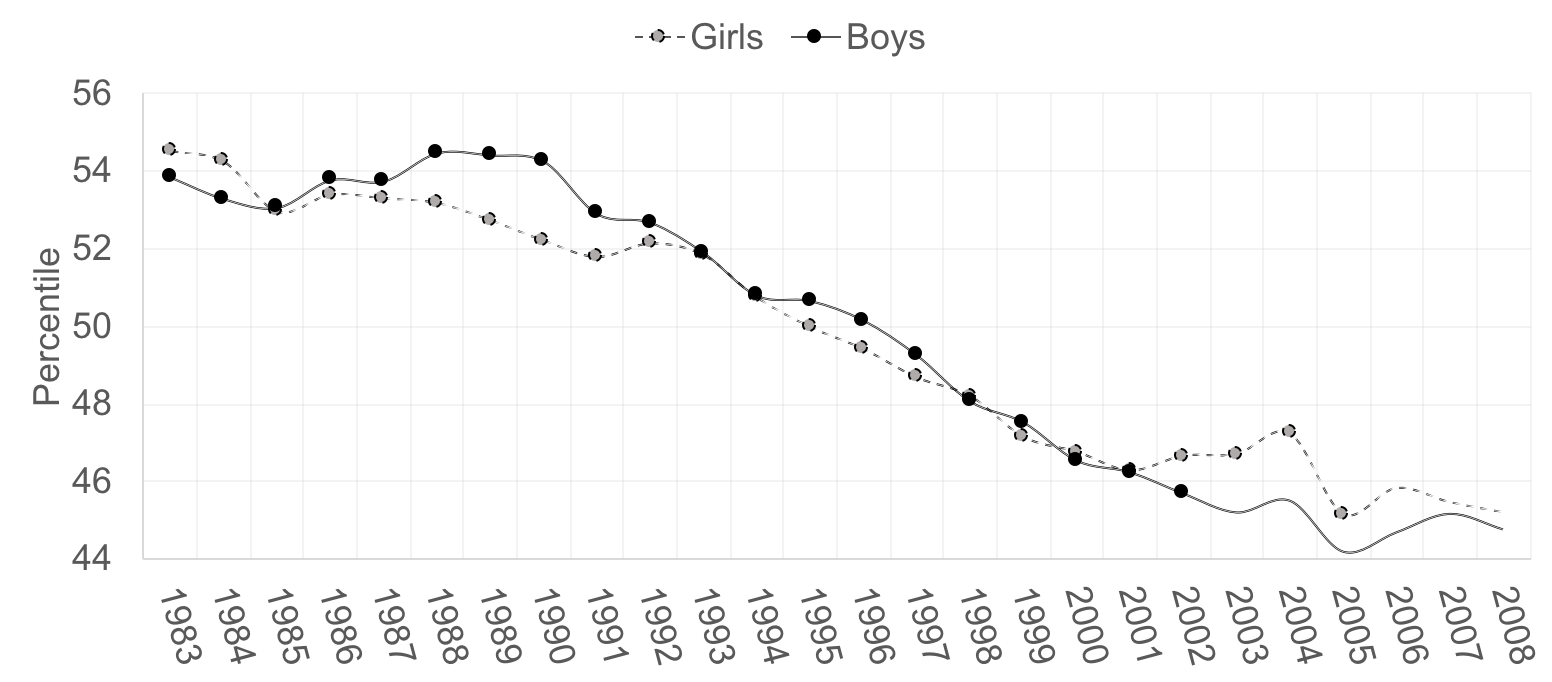


**Supplementary Figure 2.** Secular trends of the results of the standing the broad jump test by birth cohorts (2008 is the reference cohort and dots represent statistically significant differences)


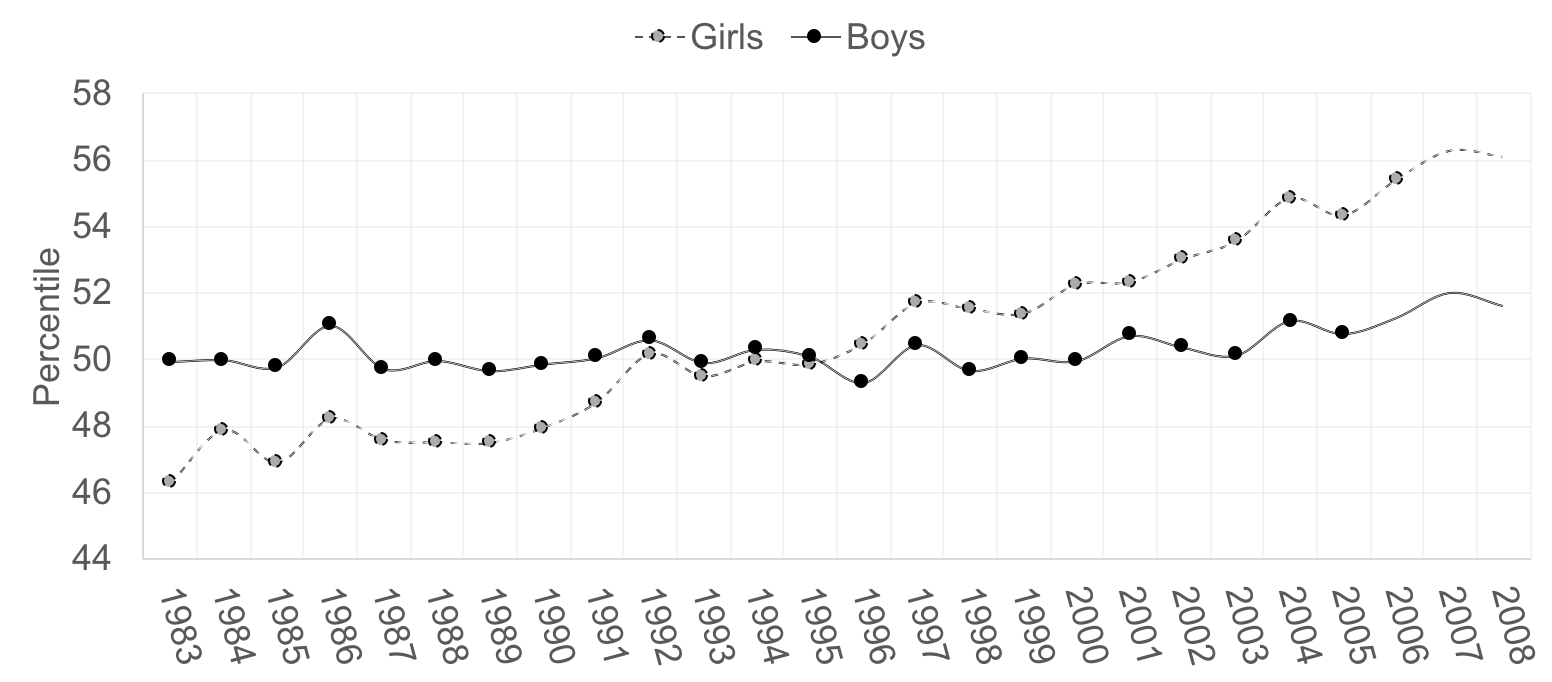


**Supplementary Figure 3.** Secular trends of the results of the backward obstacle course test by birth cohorts (2008 is the reference cohort and dots represent statistically significant differences)


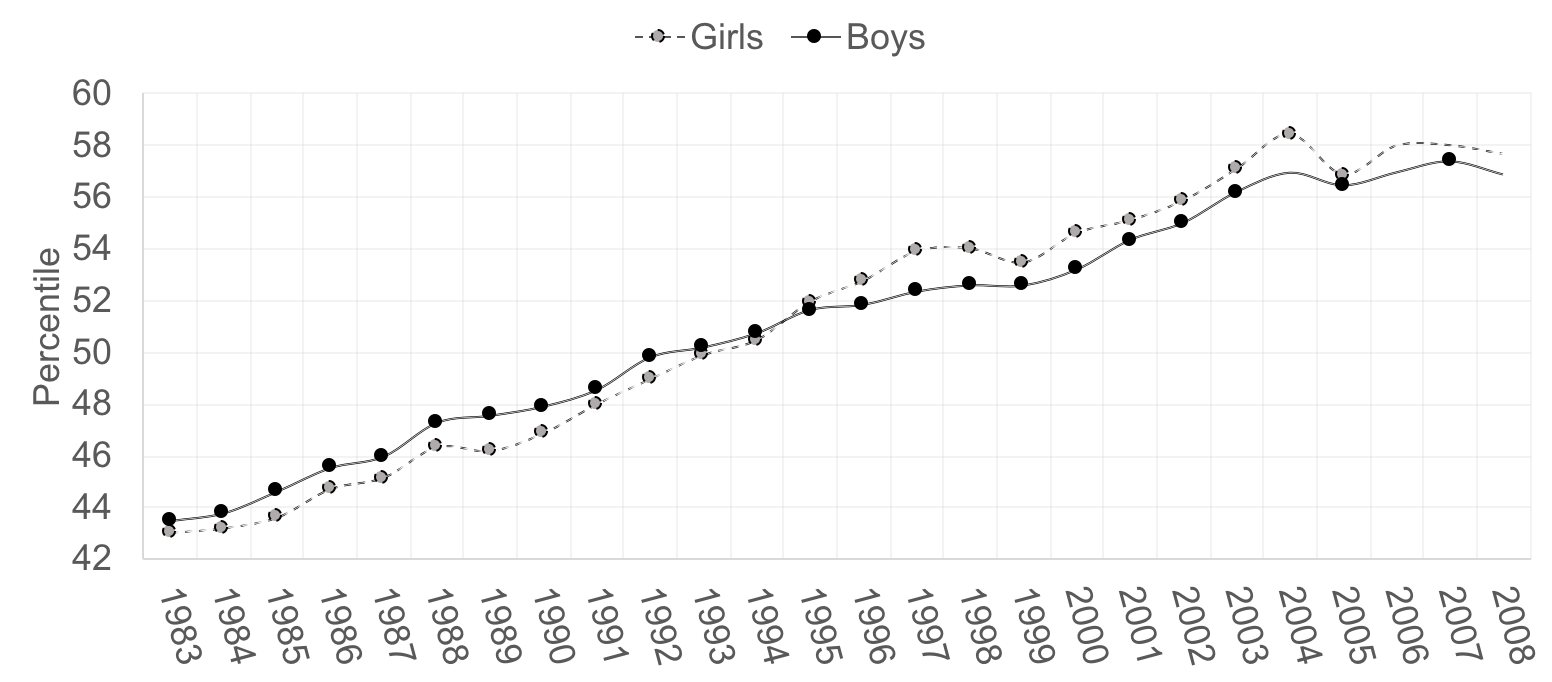
**Supplementary Figure 4.** Secular trends of the results of the one-minute sit-up test by birth cohorts (2008 is the reference cohort and dots represent statistically significant differences)


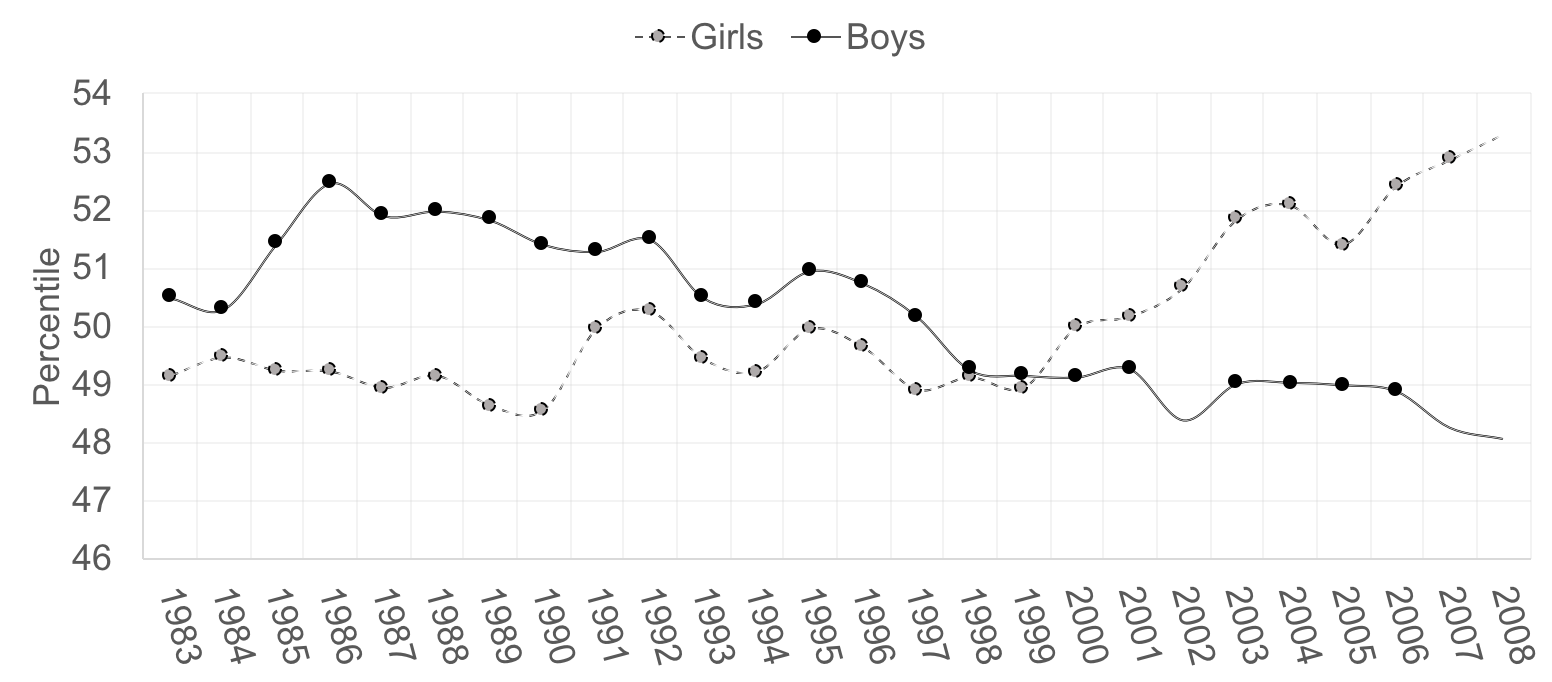


**Supplementary Figure 5.** Secular trends of the results of the stand and reach test by birth cohorts (2008 is the reference cohort and dots represent statistically significant differences)


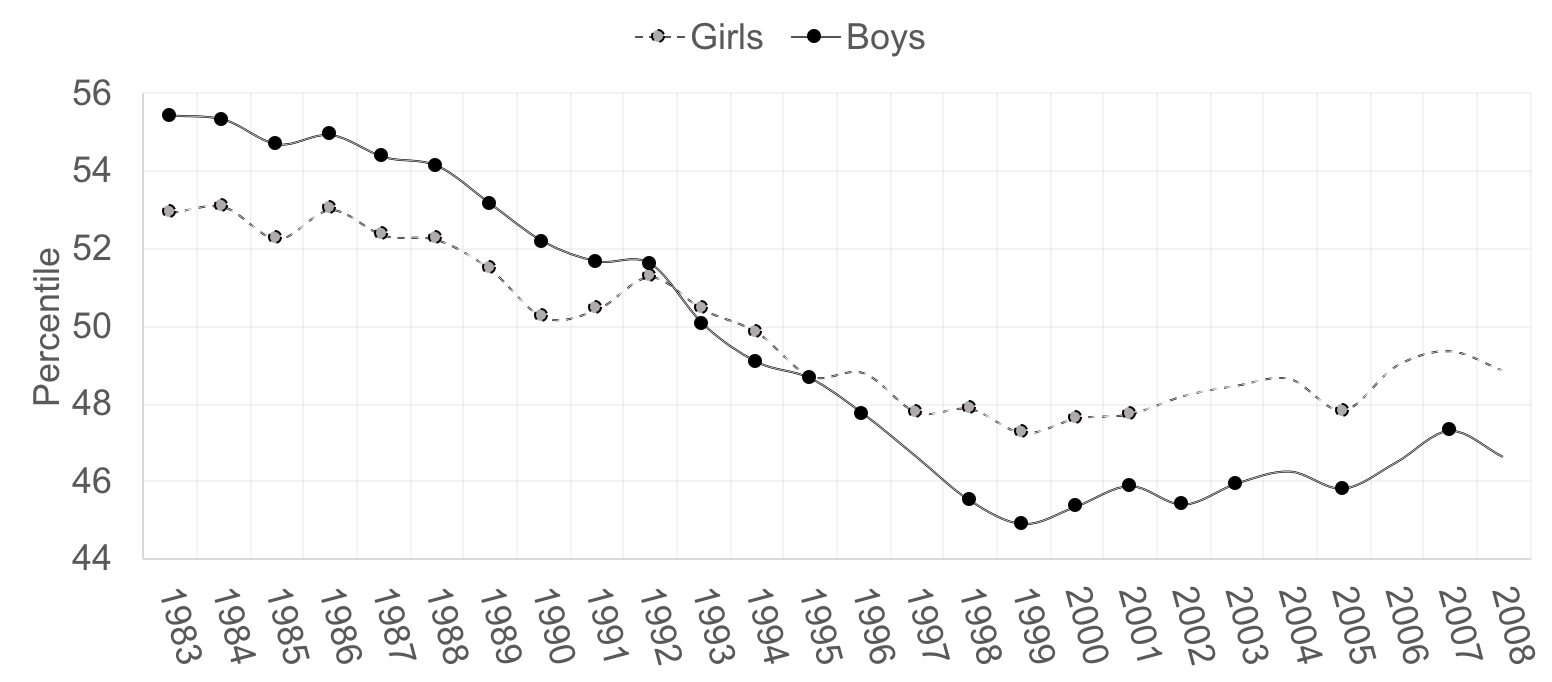


**Supplementary Figure 6.** Secular trends of the results of the bent-arm hang test by birth cohorts (2008 is the reference cohort and dots represent statistically significant differences)


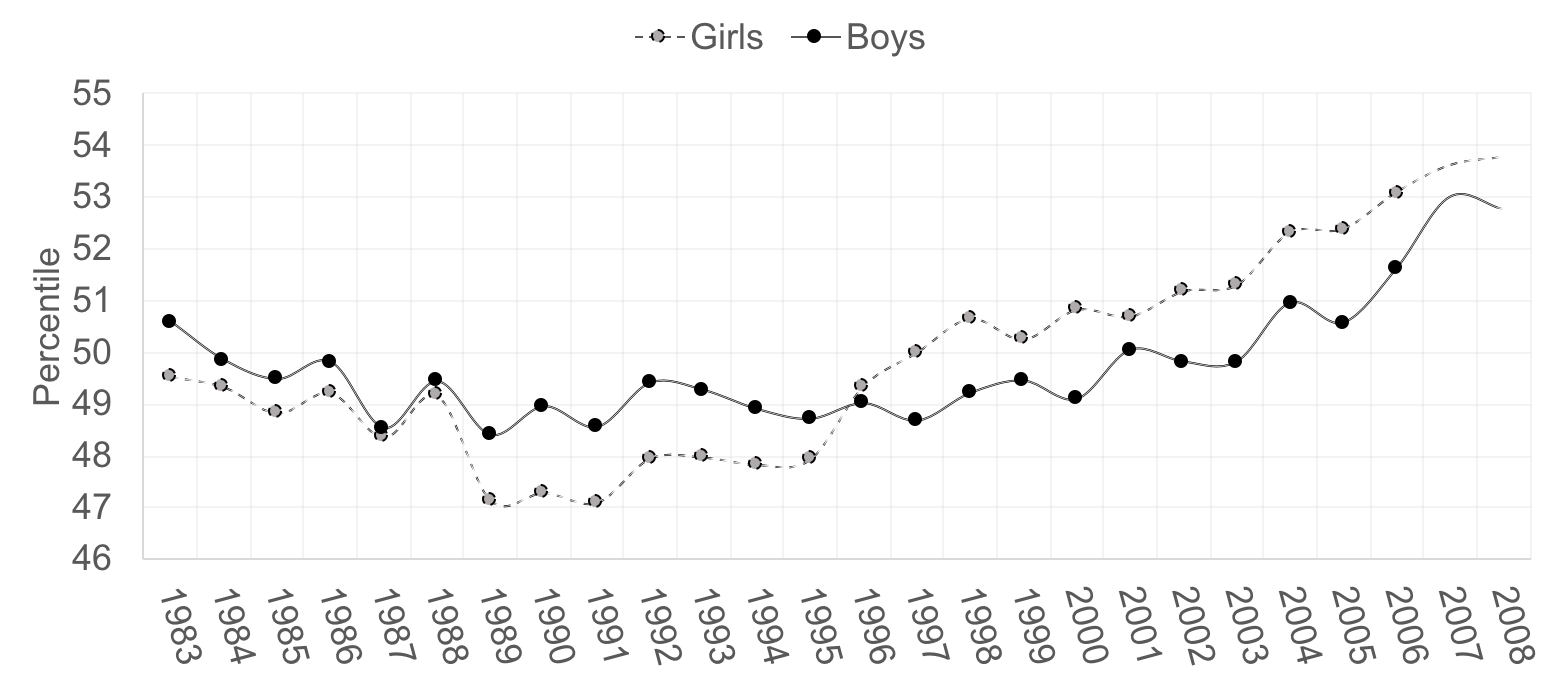


**Supplementary Figure 7.** Secular trends of the results of the 60-m dash test by birth cohorts (2008 is the reference cohort and dots represent statistically significant differences)


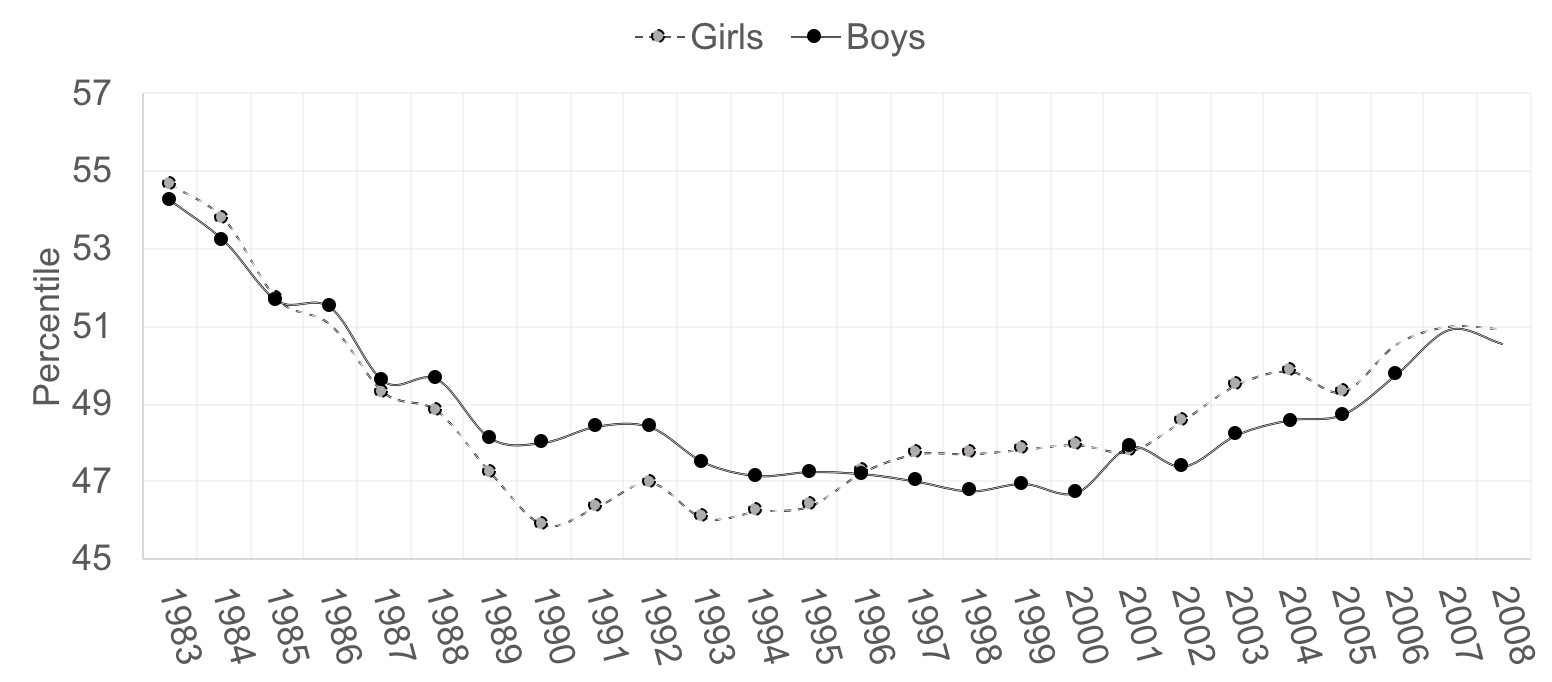


**Supplementary Figure 8.** Secular trends of the results of the 600-m run test by birth cohorts (2008 is the reference cohort and dots represent statistically significant differences)
